# Procedural Physician-Scientists as Catalysts for Innovation in Team Science and Clinical Care

**DOI:** 10.3390/cancers17152468

**Published:** 2025-07-25

**Authors:** Sajid A. Khan, Kurt S. Schultz, Nita Ahuja

**Affiliations:** 1 Department of Surgery, Yale School of Medicine, New Haven, CT 06520, USA; kurt.schultz@yale.edu; 2 Office of the Dean, University of Wisconsin School of Medicine and Public Health, Madison, WI 53705, USA

## Abstract

Procedural physician-scientists have made significant contributions to medicine and science, with twelve proceduralists receiving a Nobel Prize. Unfortunately, several systemic challenges have jeopardized the existence, let alone the flourishing, of procedural physician-scientists: the widening gap in the National Institutes of Health salary cap, decreasing funding from nonfederal public and private agencies, and shifting priorities among U.S. hospitals, payers, and policymakers toward relative value unit productivity-based compensation and fee-for-service models. Additional pressures include prolonged training pathways and the need to maintain clinical continuity. Adopting a team science approach may offer a powerful strategy to mitigate these competing demands, support rigorous scientific inquiry, and address the growing complexity of biomedical research. Concerted efforts by the federal government, policymakers, corporations, institutions, and procedural departments will also be crucial to restoring the vitality of this diminishing workforce.

## 1. Introduction

Physician-scientists are clinicians whose careers are devoted to a defined area of biomedical research conducted across academic institutions, federal agencies, private industry, and independent research centers. In addition to their scholarly endeavors, physicians-scientists provide direct patient care. By translating scientific discoveries into biomedical innovation, in addition to transforming clinical observations into research propositions, physician-scientists are invaluable members of society, serving as agents of change by mitigating disease burden for future generations.

“Procedural physician-scientists” are physicians with procedural expertise who integrate clinical practice with consistent engagement in academic pursuits, anywhere along the basic, translational, clinical, and community-based research continuum from T0 to T4 [1] Observing physiology in real time provides proceduralists with unique insights that can enrich collaborative, cross-disciplinary research and advance the objectives of team science. Procedural physician-scientists include, but are not limited to, surgeons, gastroenterologists, ophthalmologists, dermatologists, interventional cardiologists, interventional pulmonologists, and interventional radiologists.

A career as a procedural physician-scientist entails wrestling with the dynamic tension between one’s clinical aspirations and the desire never to let go of laboratory ones. By coupling hands-on skills with scientific inquiry, procedural physician-scientists embark on a lifelong journey of learning, contributing to, challenging, and advancing the field of medicine while mentoring the next generation. To have a far-reaching impact on the lives of others, one must achieve clinical excellence while also applying the utmost rigor to their research questions, and that cannot happen without a commitment to both responsibilities.

This editorial discusses the importance of procedural physician-scientists, the benefits of pursuing this career path, their impactful contributions, and their unique challenges. It also considers how departments, institutions, industry, and policymakers might address the difficulties of training and retaining future generations. All physician scientists are critical to the success of our nation’s biomedical enterprise and deserve robust support. This editorial focuses on one particularly vulnerable group: procedural physician-scientists, whose ranks are at risk of disappearing.

### 1.1. Societal Value of Procedural Physician-Scientists in Research

Procedural physician-scientists have had an illustrious record of contributions to biomedical discovery and health, as they are well-positioned to bridge the gap between laboratory discoveries in basic, translational, clinical, and population health sciences and practical applications that improve patient care [2]. Their dual expertise facilitates true bench-to-bedside research to address meaningful clinical issues, enhance evidence-based surgical practices, and drive measurable improvements in patient outcomes. They engage in the entire research life cycle. For some procedural physician-scientists, this entails testing their hypotheses in the laboratory, validating those findings in the operating room or procedure suite, and utilizing clinical specimens back in the laboratory, where the cycle continues. For others, this cycle entails in vitro testing of medical devices in animals, evaluating those devices in multi-phase clinical trials, and engaging in post-market refinements back in the laboratory.

Since 1901, twelve proceduralists have received the Nobel Prize in Physiology or Medicine [3]. In 1909, Theodore Kocher became the ninth individual and the first proceduralist to win this prestigious award. Dr. Kocher was a master general surgeon who performed 7000 thyroid operations. The most recent proceduralist awarded the Nobel Prize is Barry Marshall, a gastroenterologist, who, along with pathologist J. Robin Warren, discovered *Helicobacter pylori* and its role in gastritis and peptic ulcer disease. Other proceduralist Nobel Laureates include ophthalmologists, otolaryngologists, interventional cardiologists, orthopedic surgeons, plastic surgeons, urologists, and general surgeons.

Proceduralists have taken on significant leadership positions in the federal government, including John Niederhuber as the 13th Director of the National Cancer Institute, Monica Bertagnolli as the 17th Director of the NIH, and Martin Makary as the 27th Commissioner of the U.S. Food and Drug Administration. Procedural physician-scientists bring a unique practice-informed perspective to policymaking, drawing on their firsthand experience performing interventions that impact patient outcomes in real time. This clinical insight enables them to frame and address policy-relevant research questions with a nuanced understanding of bedside care and systems-level implications.

### 1.2. Benefits to Research Organizations of Procedural Physician-Scientists

Academic and research institutions benefit from recruiting, retaining, and promoting procedural scientists, enhancing their institution’s visibility [4]. By being situated squarely at the intersection of medicine and science, procedural surgeon-scientists are regarded as the professional role models of their field at their institution. Given the very nature of their role, they often lead national organizations, thereby improving their institution’s regional, national, and global reputation, which translates to increased procedural volume as more patients seek care at their institution.

As a procedural physician-scientist, one is equipped to generate new knowledge that influences institutional and national research priorities, paving the way for an intellectually stimulating and enriching career [5,6]. As aptly stated by Francis Collins and his co-authors in their letter in *Science,* basic science is the “engine that powers the biomedical enterprise” [7]. Traditional basic and translational research pursuits include investigating the tumor microenvironment, studying cellular or gene therapies, and analyzing the role of the gut microbiota in surgical disease [8,9,10]. On the other hand, procedural physician-“trialists” rigorously and pragmatically test new drugs, devices, and psychosocial interventions in controlled and real-world settings [11,12]. Procedural physician-scientists also conduct cost-effectiveness analyses and pursue data science and artificial intelligence techniques to derive meaning from vast amounts of unstructured information [13,14,15,16,17]. Additionally, procedural physician-scientists undertake complex mixed-methods approaches and conduct implementation science to generate hypotheses about causal mechanisms, enhance adherence to evidence-based practices, and curtail adherence to those that are not [18].

There is a well-documented positive return on investment for federal agencies, foundations, and private industry that fund the work of procedural physician scientists. In a study across the entire NIH from 1997 to 2003, the K-to-R transition rate was 30% [19]. Previous studies have reported comparable, if not slightly higher, transition rates for procedural-scientists: 39% for pediatric surgeons [20], 39% for thoracic surgeons [21]. and 35% for ophthalmologists [22]. In a 22-year analysis of the Society for Vascular Surgery Foundation Mentored Research Career Development Award, which provides supplemental funds to K-awardees, the K-to-R transition rate was impressive, at 62% [2]. This outcome translated to a 9.5-fold return on investment for the foundation that funded these procedural scientists.

### 1.3. Challenges of Being a Procedural Physician-Scientist

Physician-scientists constitute 30% of all federally funded investigators [23], whereas the surgeon-scientist workforce constitutes less than 2% [24]. Procedural physician-scientists offer a specialized scholarly perspective due to their technical clinical responsibilities but face unique demands, different than those of traditional physician-scientists. While it is challenging to determine the amount of NIH funding awarded to any proceduralists, NIH funding to surgeon-scientists has declined over time relative to their medical counterparts. This is likely due to the lower volume of surgical applications, the smaller growth in submissions, and the significantly lower success rate compared to non-surgical NIH applications [25]. This situation is made all the more harrowing because surgeons have contributed considerably to our understanding of physiology and pathology [26]. A 2024 report found that the proportion of surgeons in the U.S. with NIH funding remains <1.0% (0.5% in 2010 to 0.7% in 2020) [27].

Surgeon-scientists are an endangered species [28]. likely due to the dynamic tension they face between clinical demands, especially within procedure-based revenue models, and the challenge of securing extramural funding amidst increasingly competitive NIH funding pay lines. Surgeon-scientists encounter additional challenges throughout their careers, ranging from the limitations of the traditional surgical training paradigm to the burden of administrative responsibilities [29,30]. Disconcertingly, the current zeitgeist among surgeons reflects an erosion of research as a core professional value, as seen in declining rates of grant applications, funding success, and academic publications compared to prior decades [29,31,32].

Basic science research appears disproportionately affected by the modern challenges of succeeding as a procedural physician-scientist [32]. In a survey of 2504 academic surgeons, two-thirds said it was unrealistic to expect surgeons to conduct basic research, citing a lack of time, motivation, and departmental support [31]. In a qualitative study, K awardees cited their research years in residency as sparking their interest in a surgeon-scientist career. Nonetheless, they reported needing additional time as early-career faculty to conduct preliminary research for their K grant application. Finding time is difficult for junior surgeons with external and internal pressures to be clinically productive [33]. The interviewed surgical chairs noted that this time is more abundant for medical physician-scientists within their training paradigm [34].

The NIH salary cap gap has widened over time for surgeon-scientists, and NIH funding for surgeons has declined [24,32,35]. Procedural physician-scientists are less likely to transition to independent research funding than non-procedural scientists [36]. Funding for surgeon-initiated grants is set to decrease further with the proposed nearly $18 billion reduction or more in NIH funding under the current federal administration [37].

### 1.4. Addressing the Challenges of Training and Retaining Procedural Physician-Scientists

Hospitals in the United States increasingly depend on the clinical revenue generated by procedural physician-scientists, particularly as reimbursement rates from Medicare and Medicaid, across procedural specialties, continue to fall behind the inflation rate [38,39,40,41]. Yet, medicine relies on all physician-scientists, including those in procedural specialties, to translate scientific advances into meaningful clinical improvements, particularly given that nearly 30% of the global disease burden is amenable to surgical treatment [42]. Institutions should urge policymakers to redesign healthcare systems to incentivize proceduralists to conduct research and mitigate clinical demands [24]. At the Baylor College of Medicine, an academic relative-value unit scoring system increased departmental academic productivity [43]. Institutions should also advocate for policies that adequately compensate federally funded procedural physician-scientists while ensuring the quality of grant awardees. Simultaneously, procedural departments should adopt alternative funding mechanisms to support protected research time for procedural physician-scientists and cultivate an environment that values, awards, and promotes their contributions. Department leadership should actively encourage junior faculty to pursue mentored career development awards from the NIH and specialty societies and apply for societal and foundation awards, such as those through the American Surgical Association, American Society for Clinical Investigation, the Burroughs Wellcome Fund, and the Howard Hughes Foundation [44,45,46,47,48,49].

Specialties with a high procedural volume have varied in their efforts to invest in clinician scientists [24]. The American Academy of Otolaryngology-Head and Neck Surgery Foundation, the Triological Society, and the Society for Vascular Surgery Foundation have established collaborative investments with the NIH [50]. In 2011, the National Institute on Deafness and Other Communication Disorders established the Otolaryngology Surgeon-Scientist Program to provide training and mentorship, aiming to increase the number of otolaryngologists securing independent, tenure-track surgeon-scientist positions [51]. From 1999 to 2019, 77% of the Society for Vascular Surgery Foundation Mentored Research Career Development Awards recipients have obtained NIH R01, Veterans Affairs Merit, Department of Defense funding, or industry research funding [52]. The National Cancer Institute (NCI) has established partnerships with institutions serving underserved patient populations and trainees, as well as with NCI-designated Cancer Centers. The NCI’s Early-Stage Surgeon Scientist Program offers administrative supplemental funding to select early-stage surgeon scientists [53]. The American College of Gastroenterology supports junior faculty through career development awards. A 25-year longitudinal analysis found that 95% of awardees remained in academia [54]. Additionally, the American Association of Obstetricians and Gynecologists Foundation and the Reproductive Scientist Development Program sponsor research training fellowships. In a survey of fellowship graduates, 59% received NIH funding, and 94% reported that the fellowship was critically important for their academic careers [55].

Other professional societies and academic institutions with procedural-intensive practices should seek similar alliances with institutes and centers within the NIH. As the number of these partnerships grows, a stronger argument could be made to the NIH to establish an institute dedicated to procedural research. Regardless of whether a procedural institute is the answer, these societal-governmental relationships will hopefully increase proceduralists’ representation on the institutional councils and study sections through the Center for Scientific Review [56].

There are established mechanisms for obtaining extramural funding for junior and senior surgery faculty beyond the NIH. Within the U.S. Department of Health and Human Services, the Agency for Healthcare Research and Quality (AHRQ) offers several grant mechanisms for procedural physician-scientists conducting research in health services, patient safety, quality improvement, implementation science, and healthcare delivery [57]. However, the current federal administration has proposed $129 million in cuts to the AHRQ [37]. The Department of Defense (DoD) is another federal department that funds biomedical research. Established in 2009, the Peer-Reviewed Orthopaedic Research Program (PRORP) supports researchers seeking to combat musculoskeletal disease [58]. The current administration has proposed a $113 billion increase to the DoD budget, although it remains unclear whether research is a funding priority [37].

The U.S. Department of Veterans Affairs represents a valuable yet underappreciated platform for building a meaningful and impactful career as a procedural physician-scientist [59]. The VA’s research infrastructure and grant award mechanism, analogous to that of the National Institutes of Health, have empowered surgeon-scientists across the research continuum to thrive [26]. The VA has state-of-the-art equipment housed in funded core facilities for basic scientists. The VA’s National Surgical Quality Improvement Program provides researchers with billions of data points for health services. Lastly, the VA Cooperative Studies Program enables trialists to conduct large-scale, multicenter clinical studies [60].

A life as a procedural clinician means a career dedicated to caring for individual patients in the procedure room. In contrast, a life as a scientist means a career devoted to impacting the lives of thousands of patients in the laboratory, whom they will never meet. Whenever a procedural surgeon-scientist is in the hospital, they are not in the laboratory, and vice versa. Thus, team science is integral to the success of a procedural physician-scientist (Box 1). Cross-disciplinary collaboration is a crucible for transformative perspectives on a research problem and may translate to a higher chance of securing intramural and extramural funding.

Box 1 **Targets to Increase the Procedural Physician-Scientist Workforce to Match the Need**

                   Team Science and Mentorship

- Promote cross-disciplinary collaborations for procedure-based research teams
- Encourage procedural physician-scientist to utilize mosaic mentorship strategies
- Establish biostatistical, bioinformatics, mixed methods, clinical trial, and grant writing support
- Build shared departmental laboratory space and centralized equipment resources

             Departmental and Institutional Support—Trainee Level

- Identify early trainees with demonstrated passion for science anywhere along the T0 to T4 research continuum
- Create training programs (e.g., PSTPs) for aspiring procedural physician-scientists (Table 1)
- Accelerate the entry of proceduralists into their subspeciality of interest (e.g., Flexibility in Surgical Training)

              Departmental and Institutional Support—Faculty Level

- Develop formal mentorship-matching programs for K-grant applicants
- Institute RVU equivalents and/or awards for good mentorship and academic RVU equivalents for good science
- Provide administrative positions for procedural physician-scientists to match salaries above the NIH salary cap
- Offer startup funds and bridge funding

                 Federal Support and Policy Change

- Establish a dedicated institute for procedural research within the NIH
- Encourage more proceduralists to serve on study sections through the Center for Scientific Review
- Increase the payline for procedural physician-scientists while maintaining the rigor of surgical science
- Extend the time for surgeons to be considered early-investigators
- Build partnerships between procedural societies/foundations and NIH institutes/centers

                   Nonfederal Funding Sources

- Identify grateful patients as potential philanthropic sources
- Build partnerships between nonfederal public/private agencies and academic institutions

Recognizing the value of team science, the NIH introduced the Multiple Principal Investigators (MPI) option in 2006, enabling research teams to designate more than one program director or principal investigator [61]. In fiscal year 2022, surgeons were listed as the other principal investigators on 438 MPI grants compared to 160 awarded in fiscal year 2012 [62]. To reduce turnover and sustain momentum, leaders of transdisciplinary research teams should define concrete, shared goals, assign clear roles, offer strong support, train in conflict resolution and negotiation tactics, facilitate cross-institutional communication, convene regular team-wide meetings, and promote equitable opportunities for advancement [63].

The Yale Center for Clinical Investigation, within the Yale School of Medicine, launched the Team Science Program in 2020 [64]. to establish and support an institutional culture of collaborative research and allow for opportunities for a team science approach. Such team science programs are part of Clinical and Translational Science Award (CTSA) hubs, which are funded by the NIH and designed to accelerate interdisciplinary science. Other institutions with similar programs include the University of Pennsylvania, the University of North Carolina, Harvard, the University of California, San Francisco, the University of Michigan, Northwestern University, Washington University in St. Louis, the University of Pittsburgh, and Duke University.

Although team science is gaining recognition in many academic environments, there is still an ongoing emphasis on principal investigator and last authorship for faculty tenure and academic promotions. Offices of academic and professional development at universities need to make concerted efforts to reward team science-based research projects and promote faculty who are procedural physician-scientists. Additionally, the current indirect cost financial allocation model across individual departments undermines the value of collaborative research, disproportionately disadvantaging proceduralists and multidisciplinary teams. Mechanisms should be established at universities to enable the distribution of indirect costs among departments for team science-based federal funding. University and school level leaders at the President/Chancellor/Provost and/or Dean levels should encourage interdisciplinary partnerships and innovative funding paradigms to bridge existing gaps. Structured team science programs offer a promising pathway to revitalize the careers of procedural physician-scientists by alleviating competing demands on their time while maintaining a commitment to rigorous scientific inquiry.

Departments should actively identify early trainees with the personal qualities of a procedural surgeon-scientist (e.g., intellectual curiosity, resilience, grit, scientific rigor, patient-centeredness, team-oriented) [65], provide them with the necessary environment and infrastructure to flourish, and educate them on all the funding sources available to surgeons throughout their careers [35]. Surgeon-scientists still in training should be encouraged to apply for a grant through the NIH Loan Repayment Program, whose mission is to support and retain physician-scientists [66]. For a more longitudinal experience, Physician-Scientist Training Programs (PSTPs) are designed to provide additional structure to postgraduate trainees on their path to becoming independent investigators [67]. Internal medicine, pediatrics, dermatology, pathology, radiation oncology (i.e., Holman Research Pathway), and neurology have board-sanctioned PSTPs, which are offered at over thirty medical schools [68]. These programs integrate clinical and research training and often streamline subspecialty certification to support and sustain the career trajectory of physician-scientists. Efforts should be made to establish board-sanctioned PSTPs in more procedurally intensive specialties. In 2011, the American Board of Surgery approved a new policy for Flexibility in Surgical Training (FIT), designed to support senior residents seeking focused education in their chosen specialty of interest [69,70]. Washington University in St. Louis reported favorable outcomes from a seven-institution study examining these flexibility tracks [71,72]. Future studies should investigate whether FIT mitigates the attrition of procedural physician-scientists (Box 1).

Several nationwide departments have created surgeon scientist training programs (SSTP) for aspiring surgeon-scientists to fast-track trainees to independent funding (Table 1). A core component across these programs is longitudinal mentorship. A 2025 *Annals of Surgery* study found that only 17% of academic general surgery residency program graduates were pursuing academic careers [73]. Residents with a longitudinal research mentor had 2.2 times higher odds of pursuing an academic career. Thus, surgical departments committed to replenishing this endangered workforce should establish SSTPs. While no one-size-fits-all framework exists for these training programs, certain elements should be included to ensure their success (e.g., ample protected research time and support to aggressively apply for multiple intramural and extramural grants throughout their training period). Outside the confines of the surgery department, the National Clinician Scholars Program is a well-established, two-year, multi-institutional, interprofessional research and leadership program for physicians and PhD-prepared nurses who have recently completed their training [74]. The program makes an exception for surgery residents, of which surgery residents at any U.S. program are eligible to apply. The NCSP curriculum strongly emphasizes the importance of team science, mentorship, and community-engaged research, and, historically, it has produced a diverse group of academic surgeons [75]. Given the diverse roles of a procedural physician-scientist, mosaic mentorship—enlisting a team of mentors with complementary expertise—can maximize outcomes across their various axes of professional development [76,77].

**Table 1:** Examples of surgeon-scientist training programs in the United States.

Institution	Program Name	Year of Inception
Wake Forest University School of Medicine	Orthopedic Physician Scientist Training Program [78]	1999
Ohio State University	Research Training Program [79]	2002
Baylor College of Medicine	General Surgery Residency Research Track [80]	2008
Northwestern University	Transplant Surgery Scientist Training Program [81]	2008
	Vascular Surgeon Scientist Training Program [82]	2015
	Physician Scientist Training Program [83]	Unknown
University of California, San Diego	Surgical Oncologists as Scientists (SOAS) Program [84]	2010
Johns Hopkings University School of Medicine	Physician Scientist Training Program (PSTP) [85]	2016
Cleveland Clinic	Physician Researchers Innovating in Science and Medicine (PRISM) [86]	2017
University of Michigan	Physician Scientist Residency Training Award in Urology [87]	2017
	Cardiac Surgery Physician-Scientist Training Program [87]	Unknown
Duke University	Physician-Scientist Training Program (R38 Stimulating Access to Research in Residency [StARR] Pathway) [88]	2018
	Otolaryngology Surgeon-Scientist Career Path (OSSP) [89]	2022
Stanford University	Otolaryngology Clinician-Scientist Training Program (CSTP) [90]	2018
	Research Residency Physician-Scientist Training Program (PSTP) [91]	Unknown
Yale University	Surgeon Scientist Training Program (SSTP) [92]	2021
Mount Sinai Health System	Surgeon-Scientist Training Program [93]	2023
University of Southern California Keck School of Medicine	Physician-Scientist Training Program [94]	Unknown

## 2. Conclusions

Procedural physician-scientists play a critical role in advancing patient care and surgical innovation. However, they only represent a small proportion of NIH investigators and face a widening funding gap. Government, funders, and institutions must aggressively support, invest in, and cultivate the future generations of procedural physician-scientists. As pressure to receive grant funding, publish papers, and perform procedures persists, procedural surgeon-scientists will require mosaic mentorship and reliance on team science. The crossroads of medicine and science continue to evolve, in the face of financial and infrastructure challenges, making it even more important to advocate now for innovative approaches to preserve and uphold this dual identity.
